# Neurocysticercosis detected by targeted next-generation sequencing of cerebrospinal fluid: a case report

**DOI:** 10.3389/fneur.2025.1504348

**Published:** 2025-04-17

**Authors:** Xintong Ren, HuiQin Sun, Yanghong Cheng, Yunzhou Zhang, Daiquan Gao

**Affiliations:** ^1^Department of Neurology, Xuanwu Hospital, Capital Medical University, Beijing, China; ^2^Department of Neurology, The Fifth People’s Hospital of Huai’an and the Affiliated Huai’an Hospital of Yangzhou University, Huai’an, China; ^3^Encephalopathy Rehabilitation Department, Yixian Hospital of Traditional Chinese Medicine, Baoding, China

**Keywords:** neurocysticercosis, targeted metagenomic next-generation sequencing, seizure, cerebrospinal fluid, albendazole

## Abstract

The patient, a middle-aged male with a long history of the disease, had experienced recurrent headaches for 26 years and episodic shaking of the right limb with slurred speech for the past month. He was previously diagnosed with cerebral cysticercosis and had shown improvement after anthelmintic treatment. In recent years, he noted a resurgence of headaches. One month prior, he developed right limb shaking and occasional slurred speech. A clinical neurological examination was unremarkable, but cranial MRI and cerebrospinal fluid sequencing confirmed a diagnosis of cerebral cysticercosis. Anthelmintic treatment was administered, resulting in symptom improvement.

## Introduction

Cerebral cysticercosis is the most prevalent helminthic infection affecting the central nervous system and poses a significant public health burden in many countries. Seizures and headaches are the most common clinical manifestations. Cerebrospinal fluid (CSF) examination in patients with neurocysticercosis (NCC) is helpful for diagnosing the disease. In the setting of parenchymal lesions, CSF typically shows a mildly elevated white cell count with normal glucose and protein levels, while in the setting of arachnoiditis or ventriculitis, pleocytosis with markedly elevated protein concentrations and decreased glucose concentrations may be observed. Cell counts typically show a predominance of mononuclear cells, although neutrophils or eosinophils may also be present.

In contrast, tNGS technology can analyse DNA sequences of target pathogens from samples to help determine the presence of DNA sequences of *T. solium* for more accurate diagnosis.

Certain atypical symptoms and clinical presentations can complicate early diagnosis. The common differential diagnoses for NCC include tuberculosis, toxoplasmosis, malignancy, or pyogenic cerebral abscess. The targeted metagenomic next-generation sequencing (tNGS) not only diagnoses CNS infections caused by a range of common pathogens, but also facilitates the simultaneous measurement of resistance and virulence genes.

This article describes the first case in which tNGS technology was applied to monitor the efficacy of treatment for cerebral cysticercosis. In this case, the patient had been diagnosed with cerebral cysticercosis 20 years ago and was treated with regular anticysticercosis medication. tNGS technology was used to dynamically monitor the RPM of the pig tapeworm, which was no longer detected in the cerebrospinal fluid, which may mean that the treatment has been successful and the worms have been effectively removed.

## Case presentation

The patient is a 58-year-old male with a 26-year history of recurrent headaches, which have worsened over the past 2 years. He also reports episodic tremor in the right limb and slurred speech for the past month.

Twenty-six years ago, the patient experienced severe headaches without any apparent cause, describing the pain as intolerable. He was confined to bed all day, and when the pain intensified, he also experienced blurred vision in both eyes. However, he did not report any dizziness, nausea, vomiting, numbness in the limbs, or convulsions. His headaches were persistent, and he was diagnosed with “neurological headaches” on several occasions. The symptoms did not improve significantly with symptomatic oral medication and continued to recur. Twenty-four years ago, he was readmitted to the local hospital due to a headache. A cranial magnetic resonance imaging (MRI) scan revealed the presence of cysticercosis, and he was prescribed albendazole along with other medications to help alleviate his symptoms. The patient did not experience a recurrence of the headache for 20 years. However, 2 years ago, he presented with a recurrent headache similar to previous episodes, characterized by a comparable degree of severity. The local hospital considered the possibility of hydrocephalus and initiated a course of symptomatic treatment, which resulted in a notable improvement in the patient’s condition.

**Figure 1:**
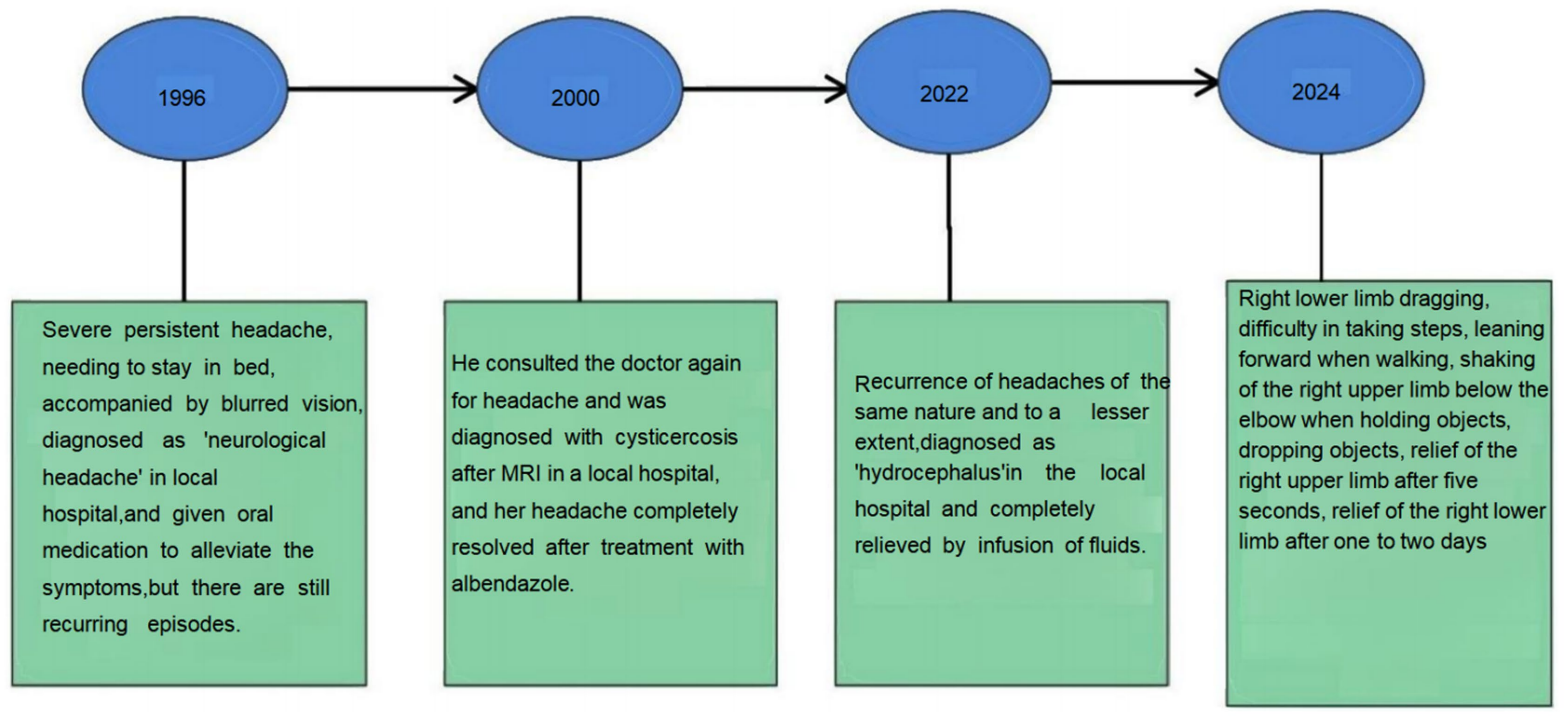
Timeline.

One month ago, the patient suddenly developed a right lower limb drag while walking, which caused difficulty in ambulation. He also experienced involuntary shaking of his right hand, lasting for about 5 s, with relief following the shaking episodes. One to 2 days after the onset of the right lower limb drag, the patient experienced episodes of shaking in the right side of the body. These episodes were accompanied by right-sided facial drooping and slurred speech, lasting about 1–5 min. After the shaking subsided, there was weakness in the right limbs; however, the patient was still able to hold objects and walk normally. He experienced one or two episodes per day. Local hospitals diagnosed him with a “cerebral infarction” and prescribed aspirin, atorvastatin, butylphthalide, and edaravone. Following this treatment, the symptoms slightly improved, with a decrease in the amplitude of the shaking and a reduction in the duration of each episode to 2–3 min. However, after discharge from the hospital, the patient continued to experience recurrent seizures, occurring once a day. He was subsequently diagnosed with ‘epilepsy’ and prescribed oxcarbazepine at a dosage of 0.15 g twice daily, which led to an improvement in his symptoms. His medical history also includes hypertension and diabetes (Figure 1).

Upon admission, the physical examination revealed the following findings: the patient was right-handed, with clear speech and normal arithmetic abilities. He exhibited hypomnesia (five words memorize zero, three words memorize zero). There were decreased visuospatial and executive abilities observed. The cranial nerve examination was negative, and limb muscle strength was graded as V, with normal muscle tone. Tendon reflexes in the extremities were ++, and there was symmetrical presence of pain and touch sensation. Coordination of movements was stable and accurate, and pathological reflexes were negative.

**Figure 2:**
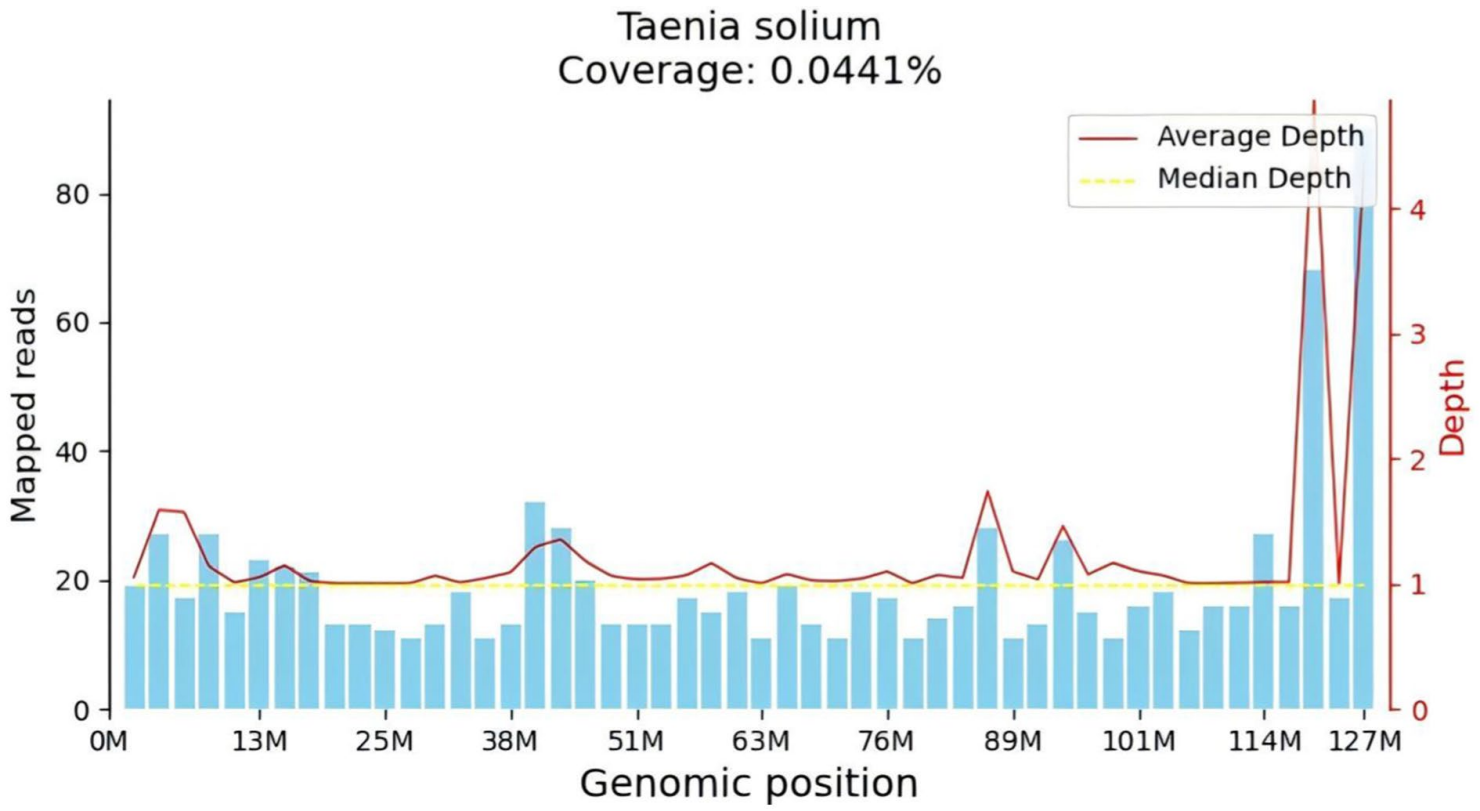
Genome mapping. *T. solium* was detected using the tNGS with coverage of 0.0441%.

After admission, the patient underwent a lumbar puncture followed by corresponding CSF tests. The lumbar puncture measured an initial pressure of 330 mmH_2_0. The routine analysis of the CSF revealed the following findings white blood cell count:70 × 10^6^/L, monocyte percentage: 75.8%, CSF protein: 94.1 mg/dL, CSF tNGS (Figure 2): *Taenia solium*, CSF immunoglobulin M: 0.69 mg/dL, CSF immunoglobulin A: 3.33 mg/dL, CSF immunoglobulin G: 34.70 mg/dL. Cerebrospinal fluid cytology revealed a large number of lymphocytes, monocytes, and neutrophils observed microscopically in the smear prepared from CSF. Cerebrospinal fluid glucose, chloride, ink stain, demyelinating antibody, autoimmune encephalitis antibody, cerebrospinal fluid smear, and cerebrospinal fluid TORCH 10 items were not abnormal. Stool routine, blood routine, biochemistry of the whole item showed no obvious abnormalities. Head-enhanced magnetic resonance imaging (MRI) showed that the left temporal lobe was markedly strengthened by a line, and the left temporal lobe sulcus appeared to be strengthened by a ring. Bilateral tibiofibular radiographs revealed small areas of increased density within the soft tissues surrounding the tibiofibular bones, which could indicate calcifications. This finding raises the possibility of a previous *Taenia solium* infection. However, it does not conclusively determine the current diagnosis.

The diagnosis were cerebral cysticercosis, symptomatic epilepsy and encephalatrophy. The patient had a history of cerebral cysticercosis and had been considered to have a ‘Herxheimer reaction’ after a significant worsening of headache symptoms at the beginning of treatment. *T. solium* was detected by CSF tNGS, and the diagnosis of cerebral cysticercosis was clear. According to the American Journal of Heat Disease (New Year’s Edition 48), Albendazole (15 mg/kg/d), praziquantel (50 mg/kg/d), and dexamethasone (0.1 mg/kg/d) were administered for 10 days, with glucocorticosteroids initiated 1 day prior to antiparasite treatment (1). The patient was administered oral albendazole at a dose of 0.2 g once daily for 3 days. Throughout the treatment, the patient experienced no exacerbation of headache, no rash, and no fever. Hence the patient was treated with albendazole at a dosage of 0.4 g three times a day orally, in combination with prednisone acetate at 50 mg once a day for a duration of 10 days. Following this period, prednisone acetate was gradually tapered off, while albendazole at 0.4 g three times a day continued for an additional 7 days. To control seizures, the patient was administered oxcarbazepine at a dosage of 0.15 g twice daily orally. Following the treatment, the patient’s condition stabilized, with no reported headaches, seizure-related limb shaking, slurred speech, or facial drooping.

In order to develop a treatment plan after discharge from the hospital, the patient underwent a repeat lumbar puncture, followed by the appropriate cerebrospinal fluid (CSF) tests. The initial pressure measured by lumbar puncture was 160 mmH_2_0. Routine analysis of the CSF showed the following results: white blood cell count: 6 × 10^6^/L, protein: 40 mg/dL, cerebrospinal fluid immunoglobulin M: 0.10 mg/dL, cerebrospinal fluid immunoglobulin A: 0.2 mg/dL, and cerebrospinal fluid immunoglobulin G: 4.60 mg/dL. The patient’s symptoms were relieved and the cerebrospinal fluid parameters returned to normal, and the patient was discharged from the hospital. After discharge, the patient continued to take oral medication under the original regimen, and was advised to undergo an outpatient review in 1 month.

After discharge, the patient continued to take oral oxcarbazepine.And prednisone acetate was tapered by 5 mg every 3 days until it was discontinued. The patient was not experiencing seizures of headache, right limb shaking and slurred speech, and discontinued oxcarbazepine on his own without further headache or seizures. The patient has not returned to the hospital for follow-up as he is asymptomatic.

## Discussion

Neurocysticercosis is a central nervous system (CNS) infection caused by the larvae (cysticerci) of the pork tapeworm, *T. solium*. It is the most prevalent helminthic CNS infection. The infection is primarily acquired through the consumption of undercooked pork containing the larvae of *T. solium*. Once ingested, the larvae can invade the digestive tract and may migrate to the brain or other tissues, where they form cysts (2). Due to its close association with the pork tapeworm, neurocysticercosis presents a significant public health challenge, particularly in regions that lack adequate meat inspection systems and effective water and food safety measures (3).

The pathogenesis of neurocysticercosis primarily involves the compression and destruction of surrounding brain tissue by cysticerci, allergic reactions and inflammation induced by antigenic proteins, and increased intracranial pressure due to the obstruction of CSF circulation pathways (4). Epilepsy and headaches are the most common clinical manifestations of neurocysticercosis. These symptoms may result from the compression or inflammation of brain tissue caused by cysticerci. The emergence of epilepsy and headaches often signifies potential neurological damage, highlighting the need for timely medical intervention to prevent further complications (5).

Diagnosing neurocysticercosis generally involves a combination of clinical symptoms, imaging examinations, and laboratory tests. Early clinical symptoms can mimic those of other neurological disorders, making the diagnosis challenging. The atypical symptoms and varied clinical presentations can further complicate early identification of the condition, underscoring the importance of a thorough diagnostic approach (6). Neuroimaging techniques, such as CT and MRI, are crucial in identifying the presence and distribution of cystic lesions in the brain associated with neurocysticercosis (7). Additionally, serological testing can identify the immune response to cysticerci in the patient, thereby confirming an active infection (8, 9). CSF examination is an important tool in diagnosing cerebral cysticercosis. In cases involving parenchymal lesions, CSF typically shows a mildly elevated white cell count with normal glucose and protein levels. Conversely, in cases of arachnoiditis or ventriculitis, pleocytosis may be present, characterized by significantly elevated protein concentrations and decreased glucose levels (10). The cell count usually reflects a predominance of mononuclear cells, although neutrophils or eosinophils may also be observed (11).

Advancements in molecular diagnostic technologies, particularly the introduction of pathogen gene sequencing, have transformed the diagnostic landscape for infectious diseases (12). The tNGS plays an important role in the diagnosis of neurocysticercosis. tNGS technology focuses on specific pathogen genes or genomic regions for deep sequencing, significantly enhancing the sensitivity and accuracy of pathogen detection, especially in cases where pathogen content is low. In the diagnosis of neurocysticercosis, tNGS can effectively detect the pathogen’s DNA (15). Compared to traditional serological tests, tNGS offers more direct evidence of the pathogen’s presence, facilitating a more reliable confirmation of the diagnosis (13). In addition, the application of tNGS technology can minimize the risk of misdiagnosis or missed diagnoses resulting from factors such as background bacterial interference and aerosol contamination. This capability enhances the accuracy and reliability of test results, making tNGS a valuable tool in the diagnostic process (1).

Del Brutto OH tapered antiepileptic drugs in 40 patients with epilepsy due to neurocysticercosis who had been free of seizures for 2 years (10). All patients previously received a course of albendazole that resulted in complete destruction of brain cysts. Multivariate analysis of the his study showed that patients who had both recurrent seizures and multiple brain cysts also had a higher risk of relapse than those with single seizures or single cysts (*p* = 0.05). This study suggests that the prognosis of epilepsy due to neurocysticercosis is not as benign as previously thought. Patients with residual calcifications and those with both recurrent seizures and multiple cysts before albendazole therapy have the highest rate of relapse after withdrawal of antiepileptic drugs. This patient was treated with albendazole 0.4 g orally three times a day plus prednisone acetate 50 mg once a day for 10 days, after which the prednisone acetate was tapered off and albendazole 0.4 g three times a day was continued for 7 days. Abraham A’s research shows that Anti-inflammatory treatment with corticosteroids in patients treated with anti-seizure medication compared to patients treated with anti-seizure medication only showed a statistically significant beneficial effect on seizure reduction (CIR 0.44, 95% CI 0.23, 0.85) and cyst resolution (CIR 1.37, 95%CI: 1.07, 1.75) (14). The results indicate that anti-seizure medication in patients with Single brain enhancing lesions neurocysticercosis whose cysts resolved can be withdrawn, while patients whose cysts calcified seem to benefit from prolonged anti-seizure medication. Additional corticosteroid treatment was found to have a beneficial effect both on seizure reduction and cyst resolution. To reduce the inflammatory response and reduce seizures, corticosteroids are used in combination during antiparasitic treatment, with specific regimens: common regimens include prednisone [1 mg/(kg·d)] or dexamethasone [0.1 mg/(kg·d)] starting at least 1 day before antiparasitic treatment.

We reported a case of a patient with neurocysticercosis who has a 20-year medical history. Utilizing the tNGS method, a substantial number of specific sequences from *T. solium* were detected. After administering standardized anti-cysticercosis treatment, the CSF was reanalyzed using the tNGS method, and no specific sequences of *T. solium* were detected. This result may indicate the success of the anti-cysticercosis treatment, suggesting effective eradication of the parasites, as no specific DNA fragments were detected to indicate their presence.

In summary, leveraging its exceptional detection capabilities, tNGS technology enables precise identification and efficacious management of patients suffering from CNS infections.

## Data Availability

The original contributions presented in the study are included in the article/supplementary material, further inquiries can be directed to the corresponding authors.

## References

[1] White AC Jr Coyle CM Rajshekhar V Singh G Hauser WA Mohanty A. Diagnosis and treatment of neurocysticercosis: 2017 clinical practice guidelines by the Infectious Diseases Society of America (IDSA) and the American Society of Tropical Medicine and Hygiene (ASTMH). Clin Infect Dis. (2018) 66:e49–75. doi: 10.1093/cid/cix1084, PMID: 29481580 PMC6248812

[2] Pineda-Reyes R White AC Jr. Neurocysticercosis: an update on diagnosis, treatment, and prevention. Curr Opin Infect Dis. (2022) 35:246–54. doi: 10.1097/QCO.0000000000000831, PMID: 35665719

[3] WHO. WHO guidelines on management of *Taenia solium* neurocysticercosis. Geneva: World Health Organization (2021). 34520139

[4] Emorinken A Erameh CO Akpasubi BO Odunlami GJ. Unmasking a hidden culprit: neurocysticercosis, an overlooked cause of acquired epilepsy. J Epilepsy Res. (2024) 14:42–6. doi: 10.14581/jer.24007, PMID: 38978528 PMC11227921

[5] Hamamoto Filho PT Norcia LF Fleury A Zanini MA. Current role of surgery in the treatment of neurocysticercosis. Pathogens. (2024) 13:218. doi: 10.3390/pathogens13030218, PMID: 38535559 PMC10976164

[6] Beatty NL Kaur H Schlaffer K Thompson K Manavalan P Rijos ZR. Subarachnoid neurocysticercosis case series reveals a significant delay in diagnosis-requiring a high index of suspicion among those at risk. Open Forum Infect Dis. (2024) 11:ofae176. doi: 10.1093/ofid/ofae176, PMID: 38680612 PMC11055394

[7] Ratcliffe C Adan G Marson A Solomon T Saini J Sinha S. Neurocysticercosis-related seizures: imaging biomarkers. Seizure. (2023) 108:13–23. doi: 10.1016/j.seizure.2023.04.005, PMID: 37060627

[8] Arroyo G Toribio L Garrido S Chile N Lopez-Urbina T Gomez-Puerta LA. Concordance between two monoclonal antibody-based antigen detection enzyme-linked immunosorbent assays for measuring cysticercal antigen levels in sera from pigs experimentally infected with *Taenia solium* and *Taenia hydatigena*. Parasit Vectors. (2024) 17:172. doi: 10.1186/s13071-024-06197-6, PMID: 38566124 PMC10988810

[9] Chile N Bernal-Teran EG Condori BJ Clark T Garcia HH Gilman RH. Characterization of antigenic proteins of the *Taenia solium* postoncospheral form. Mol Biochem Parasitol. (2024) 259:111621. doi: 10.1016/j.molbiopara.2024.111621, PMID: 38705360 PMC11197303

[10] Del Brutto OH. Diagnostic criteria for neurocysticercosis, revisited. Pathog Glob Health. (2012) 106:299–304. doi: 10.1179/2047773212Y.0000000025, PMID: 23265554 PMC4005113

[11] Garcia HH Nash TE Del Brutto OH. Clinical symptoms, diagnosis, and treatment of neurocysticercosis. Lancet Neurol. (2014) 13:1202–15. doi: 10.1016/S1474-4422(14)70094-8, PMID: 25453460 PMC6108081

[12] Mitchell SL Simner PJ. Next-generation sequencing in clinical microbiology: are we there yet? Clin Lab Med. (2019) 39:405–18. doi: 10.1016/j.cll.2019.05.003, PMID: 31383265

[13] Fei X Li C Zhang Y Zhang H Liu X Ji X. Next-generation sequencing of cerebrospinal fluid for the diagnosis of neurocysticercosis. Clin Neurol Neurosurg. (2020) 193:105752. doi: 10.1016/j.clineuro.2020.105752, PMID: 32220712

[14] Abraham A Bustos JA Carabin H de Meijere R Sahu PS Rajshekhar V. The effectiveness of anti-inflammatory and anti-seizure medication for individuals with single enhancing lesion neurocysticercosis: a meta-analysis and expert group-based consensus recommendations. PLoS Negl Trop Dis. (2021) 15:e0009193. doi: 10.1371/journal.pntd.0009193, PMID: 33788843 PMC8057605

[15] Wilson MR O'Donovan BD Gelfand JM Sample HA Chow FC Betjemann JP. Chronic meningitis investigated via metagenomic next-generation sequencing. JAMA Neurol. (2018) 75:947–55. doi: 10.1001/jamaneurol.2018.0463, PMID: 29710329 PMC5933460

